# Examining the Interface Between Substance Misuse and Intimate Partner Violence

**DOI:** 10.4137/sart.s2252

**Published:** 2009-02-18

**Authors:** Gregory L. Stuart, Timothy J. O’Farrell, Kenneth Leonard, Todd M. Moore, Jeff R. Temple, Susan E. Ramsey, Robert L. Stout, Christopher W. Kahler, Meggan M. Bucossi, Shawna M. Andersen, Patricia R. Recupero, Zach Walsh, Yael Chatav Schonbrun, David R. Strong, Emily F. Rothman, Deborah L. Rhatigan, Peter M. Monti

**Affiliations:** 1 University of Tennessee-Knoxville.; 2 Harvard Medical School Department of Psychiatry at the VA Boston Healthcare System.; 3 Research Institute on Addictions and SUNY-Buffalo.; 4 University of Texas Medical Branch at Galveston.; 5 Alpert Medical School of Brown University and Rhode Island Hospital.; 6 Pacific Institute for Research & Evaluation—Decision Sciences Institute.; 7 Brown University Center for Alcohol and Addiction Studies.; 8 Butler Hospital and Alpert Medical School of Brown University.; 9 Boston University School of Public Health.

**Keywords:** intimate partner violence, substance misuse, substance abuse, treatment

## Abstract

There is considerable theoretical and empirical support for a link between substance misuse and perpetration and victimization of intimate partner violence. This review briefly summarizes this literature and highlights current research that addresses the interface between treatment for substance abuse and intimate partner violence. Suggestions for future research and clinical implications are provided.

The prevalence and frequency of intimate partner violence (IPV) is a major public health concern. In a large study evaluating couples in the United States, over one-fifth of the sample reported experiencing IPV within the past year.^1^ Severe relationship violence, carrying a high potential for injury, is also a highly prevalent national problem, with at least 1.3 million women severely victimized annually in the United States.^2^ The economic burden of medical costs related to IPV against women in the United States is estimated to range from $2.3 billion to $7.0 billion per year.^3^ IPV constitutes an enormous problem throughout the world; a recent study conducted by the World Health Organization demonstrated that the prevalence of physical or sexual domestic violence against women ranged from 15%–71% in ten countries across the globe.^4^

IPV may be perpetrated by either gender. In a meta-analytic study, Archer^5^ found that women are slightly more likely to engage in physical IPV than men. However, two population-based studies not included in Archer’s meta-analysis demonstrated that women were more likely to report physical IPV victimization than men.^6^,^7^ Nonetheless, there is consensus that male-to-female physical violence has more destructive effects than female-to-male violence.^5^ For example, female victims of IPV are more likely than male victims to endure physical injuries and to utilize mental health and criminal justice system services.^8^–^10^ Regardless of the perpetrator gender, IPV is associated with a variety of devastating consequences, including physical injury, depression, trauma, relationship discord, divorce, suicide, and homicide.^11^,^12^

A major hurdle to studying and treating partner violence is the heterogeneity of the perpetrators and victims. Researchers have hypothesized that there may be subtypes of partner violent men or couples, with a different etiology of violence for each subtype.^13^–^16^ In light of these conceptualizations, the role of substance misuse may apply more to some subtypes of violent perpetrators or couples than to others.

There is an abundance of theoretical and empirical support for a connection between substance misuse and intimate partner violence perpetration and victimization. For example, a number of theorists have proposed etiological models of IPV in which the substance misuse of both partners plays an important role.^17^–^25^ Multivariate studies have been conducted in which these theoretical models were examined, with results showing strong empirical support.^23^,^26^–^29^

Numerous other theories have been proposed to explain the relationship between substance use and intimate partner violence perpetration. Theoretical models such as the spurious model, the indirect effects model, and the proximal effects model have been used to account for the association between IPV and substance use, with varying amounts of empirical support.^30^,^31^ In addition, researchers have examined whether certain factors may moderate the relationship between intimate partner violence and substance use.^30^,^32^,^33^ For example, researchers and theorists have suggested that the relationship between alcohol use and intimate partner violence perpetration varies according to the level of antisocial personality characteristics of the perpetrator.^30^,^32^

Empirical studies have demonstrated a strong association between the use of a variety of substances and IPV perpetration and victimization, with a majority of the research focusing on alcohol.^34^,^35^ A temporal component to substance use and IPV has also been identified, such that IPV perpetration^36^–^38^ and victimization^39^ are more likely to occur on alcohol and drug use days, relative to non-use days. Similarly, researchers have revealed a longitudinal/prospective association between substance misuse and intimate partner violence perpetration and victimization.^23^,^40^–^42^

We have been conducting cross-sectional and longitudinal research on the association between substance misuse and IPV in our laboratory for many years. In order to address enduring questions regarding the associations between substance misuse and IPV, we have, with some exceptions,^15^,^43^,^44^ employed two primary populations for these investigations that are notable for their high rates of substance misuse and violence. The first population is drawn from men and women in treatment for substance misuse. The second population is drawn from men and women arrested for domestic violence and court-referred to batterer intervention programs. A description of our central research questions, and the methods we use to address these questions in our populations of interest are provided below.

## IPV in substance abusers

A number of studies have shown that the prevalence of IPV perpetration and victimization in treatment-seeking samples of male and female substance abusers is extremely high (e.g., 50%–90% in the past year).^45^–^50^ Given extensive data showing an association between substance abuse and IPV perpetration and victimization, we have been interested in examining whether treatment for substance abuse may also bring a collateral reduction in IPV perpetration and victimization, even if the substance abuse treatment does not focus on the romantic relationship.

To test this central hypothesis, we have conducted several longitudinal studies examining the impact of treatment for substance abuse on IPV perpetration and victimization. All patients in our studies were diagnosed with alcohol abuse or dependence, and many had comorbid drug diagnoses. Substance abuse treatment involved a 5–6 day intensive outpatient partial hospitalization program, which had a cognitive-behavioral orientation administered primarily in a group format. Our research has shown significant reductions in substance use, IPV perpetration, and IPV victimization in men^51^,^52^ and women^53^,^54^ receiving treatment in an alcohol and drug partial hospital. In addition, our studies have shown that, relative to patients whose substance misuse remits, patients who relapse to substance abuse evidence greater levels of IPV perpetration and victimization. These findings are consistent with the results of other studies regarding the effects of individual treatment for substance abuse on IPV among male^55^ and female^56^ alcoholic patients.

Numerous studies have demonstrated that couples-based treatment approaches for addictions^57^ significantly reduce IPV perpetration and victimization in male^50^,^58^–^60^ and female^61^ alcoholic patients. Furthermore, in clinical trials that directly compared the effects of individually-based versus couples-based interventions for substance abuse, couples approaches have elicited superior IPV reduction outcomes.^62^–^65^

## Substance abuse in batterers

Consistent with past research, we have found that men^28^,^66^ and women^67^,^68^ arrested for domestic violence and court-referred to batterer intervention programs are at excess risk for hazardous drinking, alcohol abuse and alcohol dependence. Drug use and abuse is also highly prevalent among men^29^,^66^,^69^ and women^29^,^67^,^68^ arrested for IPV perpetration.

Research has suggested that batterer intervention programs designed to reduce IPV recidivism have poor efficacy.^70^,^71^ We have hypothesized that the lack of efficacy of these programs is partially attributable to the untreated substance abuse that is rampant among batterer intervention program participants.^72^,^73^ Indeed, men in batterer intervention programs with substance abuse evidence greater levels of violence recidivism than men with no substance use issues.^73^–^77^

In light of these data, we are currently conducting a randomized clinical trial in which hazardous drinking men arrested for domestic violence perpetration and court referred to batterer intervention programs are assigned to either a brief, motivationally focused alcohol intervention *plus* standard batterer intervention or standard batterer intervention alone. We are assessing substance use, alcohol related problems, and IPV perpetration and victimization at baseline, 3-, 6-, and 12-month follow-up. We hypothesize that adding a brief alcohol treatment to standard batterer intervention will result in less alcohol use and less IPV perpetration and victimization at all follow-up assessments, relative to standard batterer intervention alone.

Preliminary data from this study appear promising. Relative to standard batterer intervention alone, participants receiving the additional brief alcohol treatment evidence superior outcomes at one or more follow-up points in frequency of drinking, percentage of heavy drinking days, drinks per drinking day, and percentage of days abstinent from alcohol and drugs. In addition, men receiving the brief alcohol treatment have reported significantly reduced psychological and sexual aggression perpetration, relative to standard batterer intervention alone. Our research group has just begun conducting a similar randomized clinical trial with hazardous drinking women arrested for domestic violence. Our long-term goal is to establish the efficacy of the adjunct brief alcohol treatment and examine its effectiveness in reducing substance use, as well as IPV perpetration and victimization, in batterer programs across the United States.

## Future directions

Given the role of substance use in risk for IPV perpetration and victimization, there appears to be a critical role for brief interventions targeting substance use in high IPV-risk populations. Our work with individuals who have been arrested for IPV perpetration represents an early step in targeting substance use to impact IPV recidivism, and there are several other domains where similar interventions may also have a wide-reaching impact. With regard to generalizability, it would be useful to determine the extent to which the efficacy of brief interventions are limited to intimate partner violence, or whether they might transfer to other populations and other forms of interpersonal violence. For example, brief substance use interventions may be transported to populations at high risk for violence such as individuals who are incarcerated or recently released from incarceration. Additionally, substance use interventions might be effectively combined with other treatments, including parent training to more effectively decrease risk for child abuse while concurrently enhancing positive parent-child interactions. Another promising area for future research might involve increasing the specificity of our appreciation of the parameters within which brief interventions are effective for reducing violence perpetration and victimization. Specifically, several studies have determined the heterogeneity of IPV perpetrators^14^,^15^ and future studies that examine the efficacy of brief interventions across perpetrator subtypes may increase the power of treatment by facilitating the matching of treatments to perpetrator characteristics. In sum, given the preliminary evidence for the high potency of brief substance use interventions for reducing IPV perpetration and victimization, expansion and refinement of these efforts in new populations represents an important area for future research.
